# Bridging the gap: a card-based pharmacology resource to support prescribing in undergraduate dental clinical training

**DOI:** 10.3389/fmed.2026.1779768

**Published:** 2026-03-11

**Authors:** Martín Pérez-Leal, Nicla Flacco, Germán Sánchez-Herrera, Cristina Estornut, Anabel Gramatges-Rojas, Santiago Arias-Herrera

**Affiliations:** Universidad Europea de Valencia, Faculty of Health Science. Department of Dentistry, Valencia, Spain

**Keywords:** antimicrobial stewardship, dental education, just-in-time learning, microlearning, pharmacology education, prescribing confidence

## Abstract

**Background:**

In dentistry, pharmacology is usually taught during early undergraduate training, whereas prescribing decisions are mainly required later during clinical practice. This temporal gap may hinder the transfer of pharmacological knowledge to patient care and contribute to low prescribing confidence among dental students. Educational strategies that provide concise, point-of-care support may help address this challenge.

**Methods:**

A descriptive educational study using a cross-sectional survey design was conducted during undergraduate dental clinical rotations. A visual, card-based pharmacology resource based on microlearning and just-in-time learning principles (*Odontomecum in Cards*) was implemented in the clinical setting. An anonymous online survey explored students’ perceptions of the resource, including self-reported confidence in pharmacological prescribing before and after use, perceived usefulness, usability, acceptability, and qualitative feedback.

**Results:**

A total of 100 undergraduate dental students (39 fourth-year and 61 fifth-year) completed the survey. Self-reported confidence in pharmacological prescribing was higher after use of the cards in the overall sample and in both academic years. Students reported high perceived usefulness and ease of use of the resource in the clinical setting, and most indicated that they would recommend it to peers. Qualitative feedback emphasized the practicality of the cards and suggested improvements mainly related to accessibility and format.

**Conclusion:**

A visual, card-based pharmacology resource integrated into dental clinical training was well accepted by students and associated with increased perceived confidence in prescribing. These findings support the potential value of microlearning and just-in-time educational approaches in dental pharmacology education.

## 1 Introduction

Pharmacology education in dentistry is commonly delivered during the early, preclinical years of undergraduate training, whereas prescribing decisions are primarily required later, during clinical practice. This temporal and contextual separation has been identified as a key contributor to the well-documented gap between theoretical knowledge acquisition and its application in real clinical settings. In dental education, several studies have highlighted students’ difficulties in transferring pharmacological knowledge from preclinical courses to patient care, particularly during undergraduate clinical rotations (1, 2).

Safe and rational prescribing represents a complex clinical competence that extends beyond factual pharmacological knowledge, encompassing clinical judgement, confidence, and the ability to integrate patient-specific factors under time constraints. Evidence suggests that dental students and newly qualified practitioners frequently report low confidence and preparedness for pharmacological prescribing, especially in areas such as antimicrobial use and pain management (2, 3). These challenges are compounded by the high cognitive demands of clinical environments, where learners must simultaneously manage procedural tasks, patient communication, and clinical decision-making.

Concerns regarding prescribing preparedness are particularly salient in the context of antimicrobial stewardship. Dentistry accounts for a substantial proportion of community antibiotic prescriptions, and inappropriate antibiotic use in dental practice has been widely reported (4–6). Excessive duration of antibiotic treatment, incorrect drug selection, and prescribing in situations where antibiotics are not clinically indicated contribute to antimicrobial resistance and undermine global stewardship efforts. Educational interventions targeting prescribing behavior in dentistry have therefore been identified as a priority to promote safer and more rational use of medicines (4).

Traditional approaches to pharmacology education, which often rely on comprehensive manuals and didactic teaching, may be insufficient to support clinical decision-making at the point of care. In contrast, emerging educational strategies emphasize the importance of accessibility, relevance, and contextualization of learning resources. Concepts such as microlearning (delivering content in small, focused units) and just-in-time learning (providing educational support in close temporal proximity to performance) have gained increasing attention in health professions education (7, 8). These approaches are particularly well suited to clinical environments, where learners benefit from rapid access to concise, task-relevant information.

From a cognitive perspective, clinical learning is constrained by intrinsic and extraneous cognitive load, which can limit the effective retrieval and application of previously acquired knowledge. Educational resources designed to reduce unnecessary cognitive load and facilitate rapid information retrieval may therefore enhance the transfer of learning from theory to practice (8, 9). Visual and schematic formats have been shown to support learning efficiency and user engagement, particularly when integrated into authentic clinical contexts (9).

In response to the well-documented gap between early pharmacology training and its application during clinical practice, this study presents *Odontomecum in Cards*, a visual pharmacology resource designed to support rational prescribing in dentistry through a microlearning and “just-in-time” learning approach during undergraduate clinical training.

The primary objective of this study was to describe the development, educational rationale, and implementation of this card-based resource within undergraduate dental clinical training. Secondary objectives were to explore students’ perceived usefulness, usability, and acceptability of the resource during clinical rotations, and to examine self-reported changes in confidence regarding pharmacological prescribing after exposure to the cards.

## 2 Materials and methods

### 2.1 Study design

A descriptive educational study was conducted using a cross-sectional survey design. The study examined the implementation of a visual pharmacology resource (*Odontomecum in Cards*) during undergraduate dental clinical rotations and explored students’ perceptions following its use in the clinical setting. The study was approved by the Research Committee of the Universidad Europea (Approval No. 2025–810).

### 2.2 Participants

Participants were undergraduate dental students enrolled in the fourth and fifth years of the Dentistry degree program at the Universidad Europea de Valencia who were undertaking compulsory clinical rotations involving direct patient care during the 2025–2026 academic year.

All participants had previously completed the General Pharmacology course, which is taught during the second year of the program. Participation in the study was voluntary and anonymous, and no academic incentives were offered. Students were informed of the educational purpose of the study prior to completing the survey.

### 2.3 Educational resource: *Odontomecum in Cards*

*Odontomecum in Cards: Rational Use of Medicines in Dentistry* is a visual, card-based pharmacology resource designed to support rational prescribing during undergraduate dental clinical training through a microlearning and “just-in-time” educational approach. The cards were developed based on the previously published manual *Odontomecum: Basic Guide to Pharmacological Prescription in Dentistry* (10) and adapted into a concise format suitable for rapid consultation in clinical settings.

Each card summarizes prescribing-relevant information for commonly used medicines in dental practice, including the active ingredient, main indications and dosing, recommended treatment durations where applicable, contraindications and precautions, succinct antimicrobial stewardship prompts and clinically relevant interactions. The resource was conceived to facilitate point-of-care access to key pharmacological information during clinical encounters and to reinforce earlier pharmacology teaching when therapeutic decisions are required. The card-based format was intentionally designed to minimize cognitive load and enable rapid access to prescribing-relevant information during real clinical decision-making.

**Figure 1:**
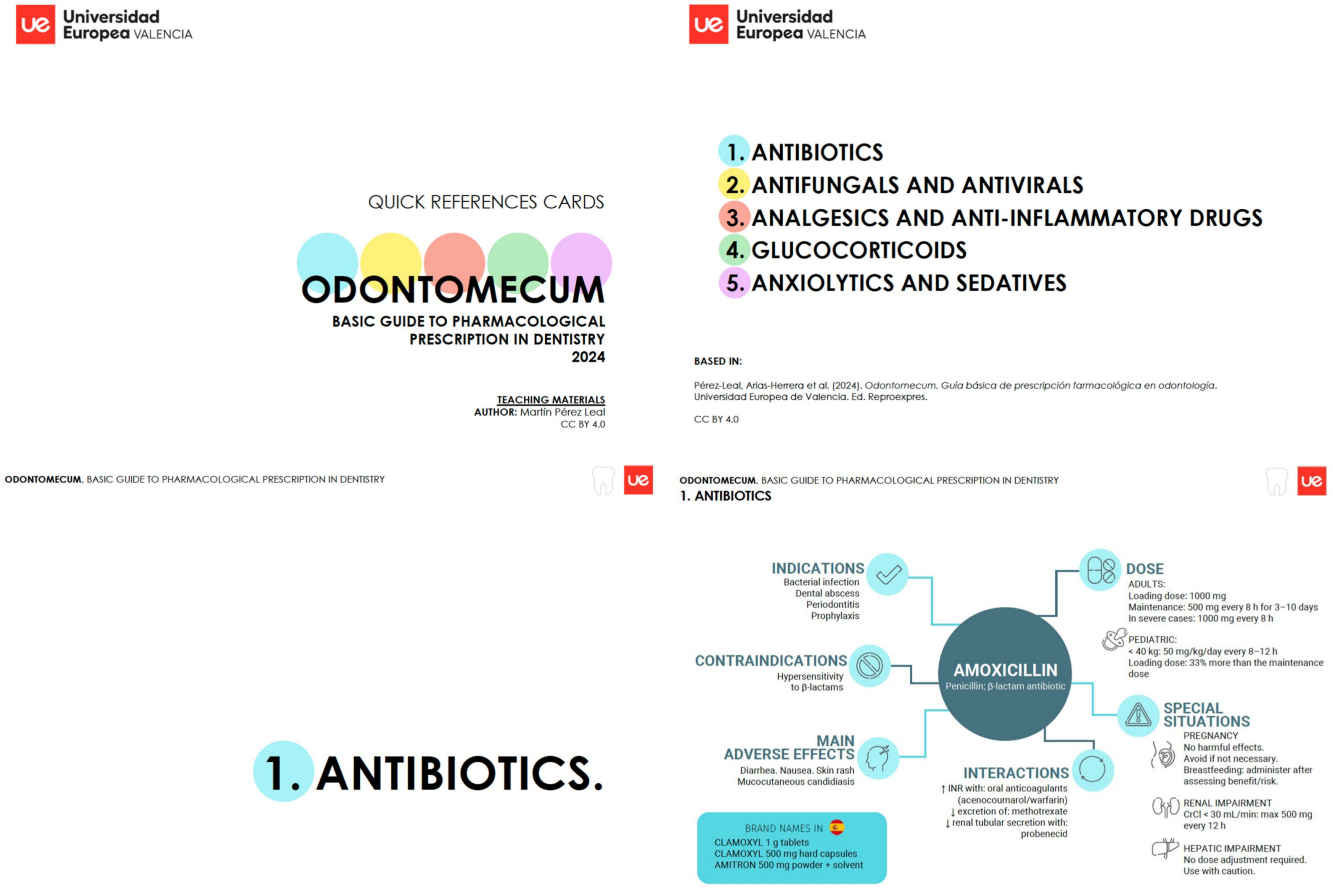
Composite graphic presenting representative pages from the pharmacological quick‑reference guide in dentistry developed at Universidad Europea de Valencia. Key sections shown include the cover, index, and an infographic example (amoxicillin) outlining indications, dosage, contraindications, adverse effects, interactions, brand names, and special considerations for pregnancy, renal and hepatic impairment.

The final version of *Odontomecum in Cards* is openly available through Zenodo (https://zenodo.org/records/17290269) (11) (Figure 1). During the study period, the cards were made accessible to students in printed format within the university dental clinics for eight weeks, coinciding with their scheduled clinical rotations. Students were informed that they could consult the cards freely during clinical practice as a support tool for prescribing-related decisions.

### 2.4 Survey instrument

Data were collected using an anonymous, online survey specifically designed for this educational study. The survey aimed to capture students’ perceptions regarding the use of *Odontomecum in Cards* during clinical practice.

The survey included Likert-scale items addressing self-reported confidence in pharmacological prescribing, with students asked to rate their confidence before and after using the cards on a five-point scale (1 = not confident at all; 5 = very confident). Additional items explored perceived usefulness of the visual format, ease of locating relevant information, frequency of use during clinical practice, perceived contribution to safer prescribing (including identification of contraindications and avoidance of prescribing errors), perceived difficulties in using the cards, and willingness to recommend the resource to peers.

In addition, the survey included a single multiple-choice item exploring students’ perceptions of the most frequent cause of inappropriate antibiotic prescribing in dentistry, included as a contextual item related to antimicrobial stewardship. An open-ended question was also included to allow participants to provide qualitative feedback and suggestions for improvement of the resource. The complete survey instrument, including all items analyzed in the Results section, is provided in Supplementary material 1.

### 2.5 Statistical analysis

Quantitative data were analyzed using GraphPad Prism (version 9.0). Continuous variables (age) are presented as mean ± standard deviation. Categorical variables are presented as frequencies and percentages.

Self-reported confidence in pharmacological prescribing was measured using a five-point Likert scale and treated as an ordinal variable. Paired comparisons of confidence before and after use of Odontomecum in Cards were performed using the Wilcoxon signed-rank test. Results are reported as median and interquartile range (IQR).

In addition to paired comparisons, changes in confidence were categorized as improved, unchanged, or worsened, based on individual differences between after- and before-use scores. These categories are presented as frequencies and percentages overall and by academic year.

Statistical significance was set at *p* < 0.05.

## 3 Results

### 3.1 Participants

A total of 100 undergraduate dental students completed the survey, including 39 fourth-year and 61 fifth-year students. Participant characteristics by academic year are summarized in Table 1.

**Table 1:** Participant characteristics and self-reported confidence in pharmacological prescribing by academic year.

		4th year (*n* = 39)	5th year (*n* = 61)	Total (*n* = 100)
Age (years), mean ± SD		23.03 ± 2.54	23.85 ± 2.18	23.53 ± 2.35
Sex, n (%)	Female	25 (64.1)	40 (65.6)	65 (65.0)
	Male	14 (35.9)	21 (34.4)	35 (35.0)
Confidence before use, median (IQR)		2 (2–3)	3 (2–4)	3 (2–4)
Confidence after use, median (IQR)		3 (3–4)	4 (3–5)	4 (3–5)
Improved, *n* (%)		26 (66.7)	45 (73.8)	71 (71.0)
Unchanged, *n* (%)		12 (30.8)	14 (22.9)	26 (26.0)
Worsened, *n* (%)		1 (2.6)	2 (3.3)	3 (3.0)
Wilcoxon signed-rank test (paired)		*p* < 0.001	*p* < 0.001	*p* < 0.001

Self-reported confidence was measured on a five-point Likert scale (1 = not confident at all; 5 = very confident). Confidence before and after use of *Odontomecum in Cards* was assessed retrospectively at a single time point.

### 3.2 Self-reported confidence in pharmacological prescribing

Self-reported confidence in pharmacological prescribing increased after use of the cards. In the overall sample (*n* = 100), the median confidence score increased from 3 (IQR 2–4) before use to 4 (IQR 3–5) after use (Table 1).

Analysis of paired ordinal responses showed that 71 students (71.0%) reported an increase in confidence, 26 (26.0%) reported no change, and 3 (3.0%) reported a decrease. Improvements were observed in both academic years, with 26 fourth-year students (66.7%) and 45 fifth-year students (73.8%) reporting increased confidence.

**Figure 2:**
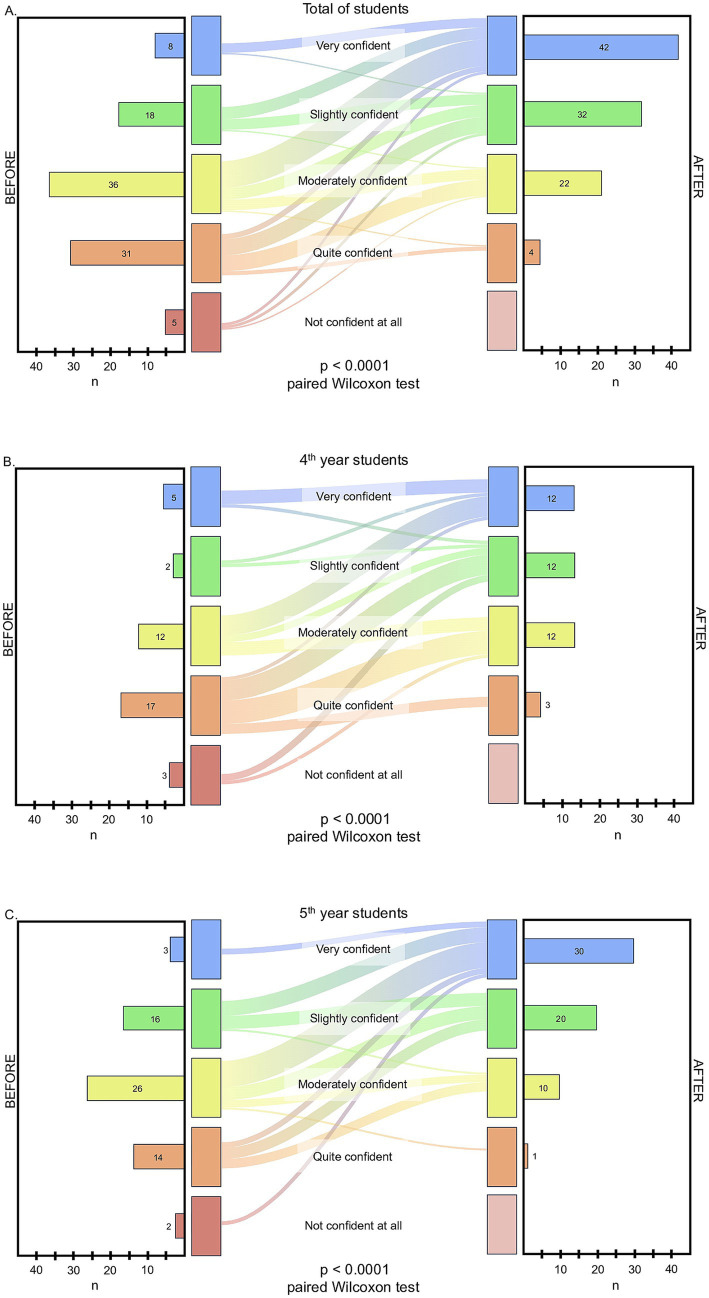
Paired transitions in self-reported prescribing confidence before and after using *Odontomecum in Cards*. Sankey diagrams show the paired flow of students across the five Likert categories from “before” (left) to “after” (right) exposure to the cards. Band thickness is proportional to the number of students; side bars display the marginal distributions. Confidence was rated on a 5-point Likert scale (1 = Not confident at all; 2 = Quite confident; 3 = Moderately confident; 4 = Highly confident; 5 = Very confident). Panels: **(A)** All students (*n* = 100); **(B)** 4th-year students (*n* = 39); **(C)** 5th-year students (*n* = 61). Paired changes in ordinal scores were analyzed using the Wilcoxon signed-rank test (*p* < 0.001).

The distribution of transitions between confidence levels before and after use of the resource is illustrated in the Sankey diagrams shown in Figure 2. The change in confidence scores was statistically significant in the overall sample and within each academic year (Wilcoxon signed-rank test, *p* < 0.001).

### 3.3 Perceived usefulness, usability, and acceptability of the resource

**Figure 3:**
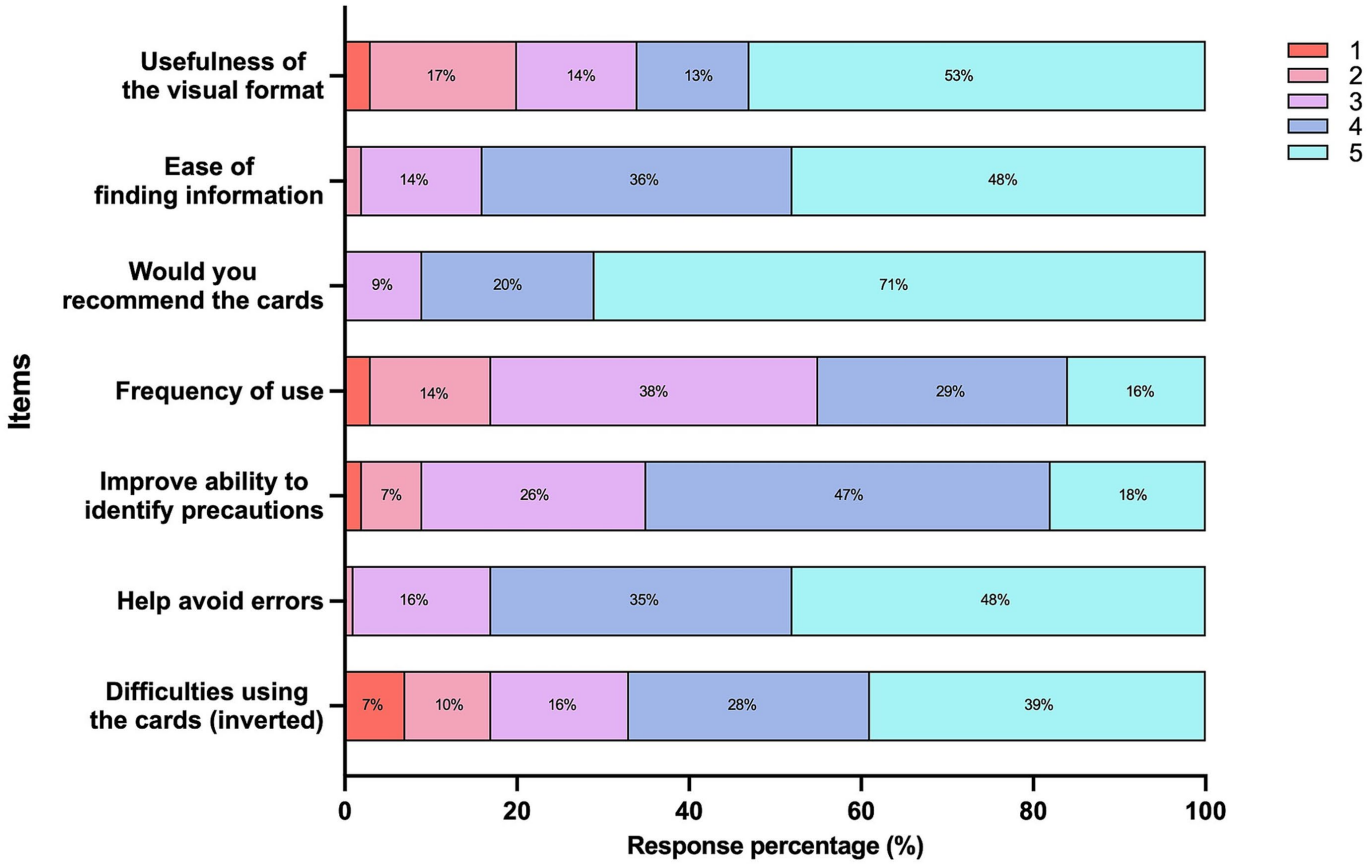
Distribution (%) of students’ responses to Likert-scale items completed after use of *Odontomecum in Cards*. Higher values indicate more favorable responses. The item “Difficulties using the cards” was reverse-coded (1 → 5) so that higher values indicate fewer difficulties. Only response categories with ≥5% of responses are shown for clarity. Full wording of the survey items is provided in Supplementary material 1.

Overall, students reported high levels of perceived usefulness and acceptability of *Odontomecum in Cards* in the clinical setting (Figure 3). Most respondents rated the visual format of the cards as quite or very useful, and indicated that relevant information was easy or very easy to locate.

Regarding frequency of use, students most commonly reported consulting the cards occasionally or frequently during clinical practice, while a smaller proportion reported use in most or almost all clinical cases. The majority of participants stated that they would recommend the resource to other students.

Perceptions of the resource’s impact on prescribing-related aspects were favorable. Many students agreed that use of the cards improved their ability to identify contraindications and precautions and helped them avoid prescribing errors and enhance patient safety. Reported difficulties in using the cards were low overall.

### 3.4 Contextual item: antibiotic prescribing

When asked about the most frequent cause of inappropriate antibiotic prescribing in dentistry, most students identified excessive treatment duration as the main contributing factor (57%). Other responses included incorrect drug choice (34%) and failure to prescribe antibiotics in severe infections (8%), while only a small proportion of students attributed inappropriate prescribing to limited antibiotic availability (1%).

### 3.5 Qualitative feedback

A total of 57 open-ended responses were analyzed. Qualitative comments were grouped into thematic categories reflecting overall positive perceptions of the resource and suggestions for improvement (Table 2).

**Table 2:** Distribution of qualitative comments by category and academic year, including anonymized representative examples.

Category	4th year (*n*)	5th year (*n*)	Total(*n*)	Representative examples
Positive comments and/or no improvements	17	21	38	“They seem very complete” “They are very helpful” “I would not change anything” “They are perfect”
More copies/availability in clinic	2	5	7	“One copy in each clinical box”
Digital access	0	3	3	“Available on the virtual campus” “Provide them digitally so we can have them on the computer”
Format-related suggestions (shorter/more schematic /visual improvements)	1	5	6	“Focus on the most relevant information” “More visible explanations” “Larger size”
Add photos of brand names	1	1	2	“Images of the commercial drug”
Comparative tables	1	0	1	“A comparative table of certain drugs”

Most comments expressed satisfaction with the content and usefulness of the cards, with many students indicating that no changes were necessary. Suggestions for improvement included increasing availability of the cards in clinical areas, providing digital access, enhancing visual design or conciseness, and incorporating brand-name references or comparative tables.

## 4 Discussion

The present study describes the development and implementation of a visual, card-based pharmacology resource designed to support undergraduate dental students during clinical practice, and explores students’ perceptions following its use. Overall, students reported increased self-confidence in pharmacological prescribing after exposure to *Odontomecum in Cards*, along with high levels of perceived usefulness, usability, and acceptability in the clinical setting.

The observed increase in self-reported confidence is consistent with previous evidence indicating that learners often feel insufficiently prepared for prescribing when pharmacology education is temporally distant from clinical application (2, 3). Rather than suggesting a direct improvement in prescribing competence, the present findings likely reflect enhanced perceived readiness and reassurance at the point of care. Confidence is a relevant educational outcome in itself, as low confidence has been associated with hesitation, overreliance on supervisors, and avoidance of prescribing decisions in clinical trainees (12).

Differences observed between fourth- and fifth-year students likely reflect varying levels of clinical exposure rather than differential effects of the resource. Fifth-year students reported higher baseline confidence, which is consistent with greater accumulated clinical experience, while both groups showed increased self-reported confidence after using the cards. This pattern suggests that the resource may be perceived as useful across different stages of clinical training, providing support both for less experienced students and for students with more advanced clinical exposure.

From an educational perspective, the positive perceptions reported by students may be interpreted in light of microlearning and just-in-time learning principles. By providing concise, task-focused information accessible during clinical encounters, *Odontomecum in Cards* may help bridge the gap between early theoretical instruction and real-world prescribing decisions. Similar educational approaches, including spaced and just-in-time learning strategies, have been shown to support knowledge reinforcement and learner engagement in pharmacology and prescribing education (7, 8, 13). The card-based format may function as a cognitive scaffold, reducing unnecessary cognitive load and facilitating retrieval of prescribing-relevant information in high-demand clinical environments (9).

To strengthen external validity and patient-safety relevance, future evaluations will incorporate objective performance measures: Objective Structured Clinical Examination (OSCE) stations focused on drug selection, dosing, contraindications, and antibiotic duration using standardized rubrics; blinded audits of student prescriptions issued during supervised rotations to quantify error rates and guideline concordance; accuracy and time-to-decision in simulated e-prescribing tasks; and study designs comparing students exposed versus unexposed to the cards.

Perceptions related to antimicrobial prescribing warrant particular attention. Although the survey item addressing antibiotic prescribing was included for contextual purposes only and did not assess objective knowledge, the fact that most students identified excessive treatment duration as a major contributor to inappropriate antibiotic use aligns with concerns reported in the dental literature (4–6). In line with this, the antibiotic cards include concise guidance on typical treatment durations and explicit prompts to review the indication and avoid unnecessary extensions, directly addressing the over-treatment pattern highlighted by students. In the next iteration, we will further increase salience (e.g., a dedicated “Duration” field and a visible “review-to-stop” cue). This finding may reflect growing awareness of antimicrobial stewardship principles among dental students and underscores the relevance of educational resources that reinforce rational prescribing during clinical training.

The qualitative feedback further supports the acceptability and perceived practicality of the resource. Students’ suggestions focused primarily on accessibility (e.g., digital availability), visual refinement, and expansion of content, rather than fundamental changes to structure or purpose. These findings suggest that the resource was well integrated into clinical workflows and perceived as complementary to existing pharmacology teaching, rather than as an additional cognitive burden.

Digital integration was a frequent request from students. In response, the possibility of complementing the printed cards with a QR-enabled, mobile-friendly repository—featuring full-text search, quick filters (e.g., by drug class, indication, contraindications), and offline PDFs for clinic workstations—is currently being considered.

Beyond dentistry, this card-based microlearning model is readily adaptable to other health professions requiring point-of-care pharmacological support (e.g., nursing, medicine, and pharmacy). Content can be rapidly tailored to discipline-specific formularies and common clinical scenarios while preserving the same visual architecture and just-in-time delivery principles.

Some limitations should be acknowledged. First, the study relied on self-reported perceptions and retrospective ratings of confidence, which may be subject to recall and response biases. Confidence was captured retrospectively at a single time point after exposure, precluding within-person pre-post comparisons and potentially inflating perceived change. Moreover, gains in self-reported confidence should not be interpreted as improvements in objective prescribing competence. Second, the survey was administered at a single institution, potentially limiting generalizability to other educational contexts. Third, the study did not assess objective prescribing competence or clinical outcomes. Consequently, no conclusions can be drawn regarding the impact of the resource on actual prescribing behavior. Future studies should therefore combine subjective perceptions with objective metrics and implement a longitudinal pre−/post−/follow-up design across multiple sites. These limitations are balanced by strengths including real-world implementation during clinical practice, a complete response rate within the sampled cohort, and the inclusion of both quantitative and qualitative data, as well as the open availability of the cards in a public repository, which enhances transparency, reproducibility, and potential adoption across institutions.

Future research should explore the impact of similar just-in-time pharmacology resources on objective prescribing outcomes, longitudinal retention, and clinical decision-making, ideally using multi-institutional designs. Integration of digital formats, such as mobile or QR-enabled access, may further enhance usability and scalability. In addition, combining perceptual measures with performance-based assessments would allow a more comprehensive evaluation of educational effectiveness.

## 5 Conclusion

*Odontomecum in Cards* represents a feasible and well-accepted educational strategy to support pharmacological prescribing during undergraduate dental clinical training. While the findings primarily reflect students’ perceptions, they highlight the potential role of microlearning and just-in-time approaches in bridging the gap between pharmacology education and clinical practice in dentistry. Next steps will prioritize objective validation of prescribing performance and the deployment of a digital layer to enhance usability and scalability.

## Data Availability

The raw data supporting the conclusions of this article will be made available by the authors, without undue reservation.
