# Supplementary material for: Bridging the gap: a card-based pharmacology resource to support prescribing in undergraduate dental clinical training
Source: Front Med (Lausanne). 2026 Mar 11;13:1779768. doi: 10.3389/fmed.2026.1779768 (PMC13013055; doi:10.3389/fmed.2026.1779768)
Supplement: Supplementary file 1 [file Data_Sheet_1.pdf]

Supplementary Material 1

Survey on the use of *Odontomecum in Cards*

The following questionnaire was administered online to undergraduate dental students after their exposure to *Odontomecum in Cards* during clinical rotations. All items were completed anonymously and at a single time point.

Demographic data

1. Year
   (4th year; 5th year)
2. Sex
   (Male; Female; Prefer not to answer)
3. Age (years): ___

Confidence in prescribing

4. Before using the cards, how would you rate your confidence in prescribing medications in dentistry?
   (1 = Not confident at all; 5 = Very confident)
5. After using the cards, how would you rate your confidence in prescribing medications in dentistry?
   (1 = Not confident at all; 5 = Very confident)

Usefulness and ease of use

6. Do you consider the visual format of the cards useful for reviewing pharmacology concepts?
   (1 = Not useful at all; 5 = Very useful)
7. Was it easy for you to find the necessary information in the cards?
   (1 = Not easy at all; 5 = Very easy)

Recommendation and frequency of use

8. Would you recommend the use of these cards to other students?
   (1 = I would not recommend it; 5 = Strongly recommend)
9. How often did you consult the cards during your clinical practice?
   (1 = Never; 2 = Rarely; 3 = Sometimes; 4 = Frequently; 5 = In almost all cases)

Perceived impact

10. Do you think using the cards has improved your ability to identify contraindications and precautions in prescribing?
    (1 = Not improved at all; 5 = Highly improved)
11. Do you consider that the cards helped you avoid prescription errors and improve patient safety?
    (1 = Strongly disagree; 5 = Strongly agree)

Difficulties

12. Did you have any difficulty using the cards during clinical practice?
    (1 = None; 5 = Many difficulties)

Contextual item: antibiotic prescribing

13. One of the most frequent reasons for inappropriate antibiotic prescription in dentistry is:

- Wrong drug choice
- Excessive treatment duration
- Lack of prescription in severe infections
- Limited availability of antibiotics

Open-ended question

14. What aspects would you improve or add to the *Odontomecum* cards?
    (Open-ended response)

All items reported in the Results section correspond exactly to the questions presented in this survey.
